# AtMAD: *Arabidopsis thaliana* multi-omics association database

**DOI:** 10.1093/nar/gkaa1042

**Published:** 2020-11-21

**Authors:** Yiheng Lan, Ruikun Sun, Jian Ouyang, Wubing Ding, Min-Jun Kim, Jun Wu, Yuhua Li, Tieliu Shi

**Affiliations:** Key Laboratory of Saline-alkali Vegetation Ecology Restoration, Ministry of Education, Northeast Forestry University, Harbin, Heilongjiang 150040, China; The Center for Bioinformatics and Computational Biology, Shanghai Key Laboratory of Regulatory Biology, the Institute of Biomedical Sciences and School of Life Sciences, East China Normal University, Shanghai 200241, China; The Center for Bioinformatics and Computational Biology, Shanghai Key Laboratory of Regulatory Biology, the Institute of Biomedical Sciences and School of Life Sciences, East China Normal University, Shanghai 200241, China; The Center for Bioinformatics and Computational Biology, Shanghai Key Laboratory of Regulatory Biology, the Institute of Biomedical Sciences and School of Life Sciences, East China Normal University, Shanghai 200241, China; The Center for Bioinformatics and Computational Biology, Shanghai Key Laboratory of Regulatory Biology, the Institute of Biomedical Sciences and School of Life Sciences, East China Normal University, Shanghai 200241, China; Key Laboratory of Saline-alkali Vegetation Ecology Restoration, Ministry of Education, Northeast Forestry University, Harbin, Heilongjiang 150040, China; The Center for Bioinformatics and Computational Biology, Shanghai Key Laboratory of Regulatory Biology, the Institute of Biomedical Sciences and School of Life Sciences, East China Normal University, Shanghai 200241, China; Key Laboratory of Saline-alkali Vegetation Ecology Restoration, Ministry of Education, Northeast Forestry University, Harbin, Heilongjiang 150040, China; The Center for Bioinformatics and Computational Biology, Shanghai Key Laboratory of Regulatory Biology, the Institute of Biomedical Sciences and School of Life Sciences, East China Normal University, Shanghai 200241, China; Big Data and Engineering Research Center, Beijing Children's Hospital, Capital Medical University, National Center for Children's Health, Beijing 100045, China

## Abstract

Integration analysis of multi-omics data provides a comprehensive landscape for understanding biological systems and mechanisms. The abundance of high-quality multi-omics data (genomics, transcriptomics, methylomics and phenomics) for the model organism *Arabidopsis thaliana* enables scientists to study the genetic mechanism of many biological processes. However, no resource is available to provide comprehensive and systematic multi-omics associations for Arabidopsis. Here, we developed an *Arabidopsis thaliana* Multi-omics Association Database (AtMAD, http://www.megabionet.org/atmad), a public repository for large-scale measurements of associations between genome, transcriptome, methylome, pathway and phenotype in Arabidopsis, designed for facilitating identification of eQTL, emQTL, Pathway-mQTL, Phenotype-pathway, GWAS, TWAS and EWAS. Candidate variants/methylations/genes were identified in AtMAD for specific phenotypes or biological processes, many of them are supported by experimental evidence. Based on the multi-omics association strategy, we have identified 11 796 *cis*-eQTLs and 10 119 *trans*-eQTLs. Among them, 68 837 environment-eQTL associations and 149 622 GWAS-eQTL associations were identified and stored in AtMAD. For expression–methylation quantitative trait loci (emQTL), we identified 265 776 emQTLs and 122 344 pathway-mQTLs. For TWAS and EWAS, we obtained 62 754 significant phenotype-gene associations and 3 993 379 significant phenotype-methylation associations, respectively. Overall, the multi-omics associated network in AtMAD will provide new insights into exploring biological mechanisms of plants at multi-omics levels.

## INTRODUCTION

As a model plant, *Arabidopsis thaliana* is widely used in multi-level genetic researches and shows an excellent feasibility for conducting genotype–phenotype association studies (1–8). The 1001 Genomes Project of *A. thaliana* have generated multi-omics data (e.g. genome, transcriptome, methylome and phenome) of large-scale *Arabidopsis* ecotypes to study the genetic mechanism of many complex traits, and association analysis strategies have been applied to identify genomic markers which cause phenotypic differences (9–13). Several GWAS-based databases based on 1001 Genomes Project have been launched, and are mainly focused on the associations between genomics and phenomics, such as AraGWAS Catalog (2,3), which was developed to elucidate genotype–phenotype relationships on population level, and CLIMtools (14) which studied environment × genome × phenotype associations in *Arabidopsis*. However, no database is available to study multi-omics associations in population scale and provide comprehensive repository of genome × transcriptome × methylome × environment × phenotype interactive network.

Recently, the 1001 Genomes Project has revealed genetic basis of quantitative variation among natural *Arabidopsis*, and provided not only detailed genomes, methylomes and transcriptomes from >1000 accessions but also geographic locations of individual ecotypes (9). As more and more phenotypic data have been generated under different public studies, AraPheno has been recently constructed to store different phenotypes ranging from flowering time to ion concentrations for 1001 project plants (15). These information enables us to explore how genetic molecules affect plant phenotypes at multiple conditions. For this purpose, we used multiple statistical approaches, including expression Quantitative Trait Loci (eQTL), expression-methylation Quantitative Trait Loci (emQTL), Genome-Wide Association Study (GWAS), Transcriptome-Wide Association Study (TWAS) and Epigenome-Wide Association Study (EWAS) to investigate the relationships between different levels of biological signals, and then constructed AtMAD to integrate various information about interactions between multi-omics in *Arabidopsis*.

AtMAD is freely available at http://www.megabionet.org/atmad and is convenient for browsing, searching and downloading data of multi-omics associations in *Arabidopsis*. Here, eQTL links genomic variation and gene expression, emQTL links methylation level and gene expression, GWAS associates genomic variation and phenotypes, while TWAS connects gene expression and phenotypes, EWAS associates methylation level and phenotypes. Moreover, we also included environmental factors and metabolic pathways in the association analysis. AtMAD is the first database to provide multi-level interactions between genome, transcriptome, methylome, environment, pathway and phenotype in *Arabidopsis* and allow researchers to identify variants, genes or methylations which are associated with specific phenotypes, environments and metabolic pathways. We believe that AtMAD provides valuable multi-omics associations and will greatly facilitate the researches of molecular genetics in *Arabidopsis*.

## MATERIALS AND METHODS

### Data collection

The genotype data of 1135 naturally inbred lines of *Arabidopsis thaliana* were downloaded from the 1001 Genomes Consortium (http://1001genomes.org/data-center.html), the RNA-seq profiling and methylation data were obtained from 1001 Epigenomes Project genome browser (http://neomorph.salk.edu/1001.php). In this study, we considered 620 accessions for which RNA-seq data, genotyping data and methylation data are available. All sequence data were derived from the tissue of 10 rosettes just before bolting.

### Phenotypes, environments and metabolic pathways

All phenotypic information was collected from AraPheno (a public database collection of *A. thaliana* phenotypes, download date: 2020-1), including 22 studies and 462 published continuous phenotypes. Geographic information of each accession was collected from 1001 Genomes resource. Climate and elevation data were obtained from WorldClim 2 (16), the recent data were extracted from the Current Conditions Bioclim rasters and sourced from the 30 arc-second rasters, with 2.5 arc-minute rasters as a fallback if collection locations fell between raster cells, and 19 bioclimatic variables were included. Pathway activities were calculated with DESeq2 normalized read count for each pathway from the latest AraCyc15.0 (*A. thaliana* col), as described by Zhang *et al.* (17).

### eQTLs, environment-associated eQTLs, GWAS-related eQTLs

The variant annotated SnpEff VCF file and imputed SNP matrix were downloaded at http://1001genomes.org/data/GMI-MPI/releases/v3.1/, and genomic variants with minor allele frequency (MAF) ≥1% were retained. As the *Arabidopsis thaliana* plant self-fertilizes, the genome of each strain can be considered as a haplotype sequence (4). Gene expression was quantified for Araport 11 annotated genes and batch normalized with the RUVseq package (under series GSE80744), genes that were expressed in fewer than 100 (15%) of the samples were removed for the eQTL analysis, and a gene was considered expressed in a sample if its read count was greater than or equal to 6 (18).

We then used the R MatrixeQTL package (19) to calculate eQTLs with an additive linear model. To remove confounding effects of population structure, we used smartpca in the EIGENSOFT program (20) to perform principal component analyses and selected the top five PCs in genotype data as covariates. To remove the batch effects of the expression data, we included 21 reference genes from previous study (21) into covariates. Variants with false discovery rates (FDR) <0.05 were defined as eQTLs. *cis*-eQTLs were defined if the SNP was within 1 Mb from the gene transcriptional start site (TSS), and *trans*-eQTLs were defined if the SNP was beyond that region.

To identify environment-associated eQTLs, we examined the associations between eQTLs and local environment of plant habitats. A linear mixed model GEMMA (version 0.98.1) (22) was used to test the correlation between eQTLs and environmental gradients. For the linear mixed model option, we used Wald test (default) to test the significant associations between eQTLs and environment. *P*-values were adjusted for multiple testing using Benjamini–Hochberg correction.

To discover GWAS-related eQTLs, we collected standardized GWAS data (with Bonferroni threshold) from the AraGWAS Catalog (download date: 2020-1) and calculated linkage disequilibrium (LD) in PopLDdecay (version 3.41) tool (23) between eQTLs and GWAS loci. eQTLs that overlap with GWAS tagSNPs and LD SNPs (*r*^2^ ≥ 0.5) were identified as GWAS-related eQTLs. All GWAS data in AtMAD were computed based on the latest imputed genotype release of all 1001 accessions using a standardized GWAS pipeline (2) and downloaded from AraGWAS Catalog under their defined Bonferroni threshold.

### emQTL

All MethylC-seq data (620 accessions) was downloaded through 1001 Epigenomes Project genome browser (http://neomorph.salk.edu/1001.php) under series GSE43857 (11). Methylation levels were calculated as the frequency of C base calls at C positions divided by the frequency of C and T base calls at C positions. Cytosine positions with at least five bases coverage were examined for differential methylation. Differentially methylated sites (DMSs) were identified by root mean square tests with false discovery rate (FDR) at 0.001, using 1000 permutations for CG, CHG and CHH context. Here, methylation site with minimum allele frequency > 0.2 were retained.

emQTL was first defined by Fleischer *et al.* (24). We calculated Pearson correlation between each methylation site and all genes. Methylation-gene pairs with Bonferroni corrected *P*-value <0.05 and |Pearson *r*| > 0.3 were considered as significant emQTL.

### TWAS

Transcriptome-wide association study associates gene expression with trait/phenotype. Gene expression was quantified for Araport 11 annotated genes and batch normalized by the RUVseq package (under series GSE80744). For 462 published phenotypes in 22 studies, we calculated Pearson correlation and Spearman correlation between each phenotype with normalized continuous values and all genes. Phenotype-gene pairs with Benjamini–Hochberg corrected *P*-value <0.05 (including both Pearson and Spearman), |Pearson *r*| > 0.3 and |Spearman *r*| > 0.3 were considered as significant results.

### EWAS

Epigenome-wide association study identifies the associations between DNA methylation levels and complex phenotypes. Cytosine positions were filtered as stated above. For 462 published phenotypes in 22 studies, we calculated Pearson correlation and Spearman correlation between each phenotype with normalized continuous values and all methylation loci. Phenotype-methylation pairs with Benjamini–Hochberg corrected *P*-value <0.05 (including both Pearson and Spearman), |Pearson *r*| > 0.3 and |Spearman *r*| > 0.3 were considered as significant results.

### Pathway-mQTLs and phenotype-pathway associations

In AtMAD, we treated each pathway as a specific molecular phenotype (25). To identify the potential epigenetic variants that are associated with each pathway, we calculated Pearson correlation and Spearman correlation between each pathway activity with normalized continuous values and all methylation loci. Pathway-mQTLs pairs with Benjamini–Hochberg corrected *P*-value <0.05 (including both Pearson and Spearman), |Pearson *r*| > 0.2 and |Spearman *r*| > 0.2 were considered as significant results. Similarly, we identified phenotype-pathway associations using both Pearson and Spearman correlation tests with threshold ‘Benjamini–Hochberg corrected *P*-value <0.05 (including both Pearson and Spearman), |Pearson *r*| > 0.3 and |Spearman *r*| > 0.3’.

### Data integration and interactive-network construction

To comprehensively explore information of phenotype-related genes, we collected multiple literature-supported gene–phenotype association data. (i) We added experiment-based gene–phenotype associations to AtMAD, all associations were confirmed by various experimental techniques (such as RNA interference, CRISPR/Cas system, etc.) and collected from the latest AtPID database (1,26,27). In parallel, the morphological images of gene-knockout mutants were also collected into AtMAD. (ii) We added associations between CNVs and AraPheno-phenotypes to AtMAD, gene statuses (gain, loss or no change) caused by large indels (50–499 bp) or CNVs (500 bp and larger) were associated to phenotypes and collected from a recent GWAS research (28).

All multi-omics associations were integrated into a point-to-point network. The interactive network included the associations of eQTLs, emQTLs, pathway-mQTL, GWAS, TWAS, EWAS, phenotype-pathway and was visualized with tool echarts in JavaScript. The interactive sub-network visually displays multiple association information for variants/phenotype/gene/pathway of interest, and can assist researchers to reveal the underlying molecule mechanisms for the formation of complex phenotypes.

### Database implementation

All data in AtMAD are stored and managed in MySQL (Version: 5.7.17). The web interface was implemented using HTML5 and PHP (version: 7.0.12), also JavaScript was used for data visualization. The service of AtMAD was deployed in Apache web server which runs on the CentOS 6.5 system. Data analyses were mainly carried out using R and Python script.

## DATABASE CONTENT AND USAGE

### Database overview

**Figure 1.**
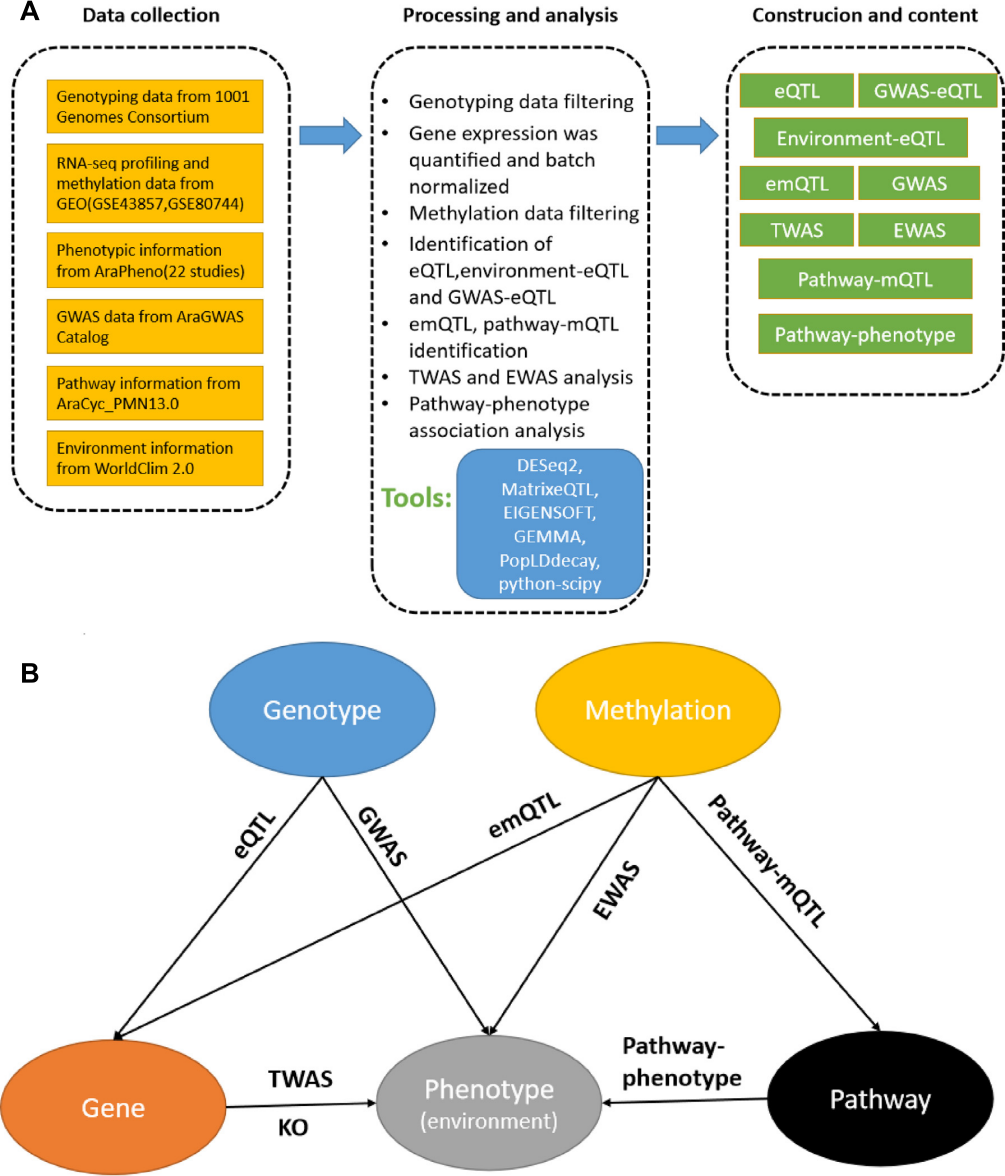
Overview of AtMAD platform. (**A**) Data collection, processing and database construction. (**B**) Schematic diagram of association network which included genotype, gene, methylation, phenotype (including environment) and pathway.

AtMAD collected the genotype, RNA-Seq, methylation and phenotype information from 1001 Genomes as well as local environment and pathway information. Data processing and analyses were performed with a standardized pipeline (Figure 1A). AtMAD contained 11 796 *cis*-eQTLs and 10 119 *trans*-eQTLs, involving totally 3879 distinct genes. Among them, 68 837 environment-eQTL associations and 149 622 GWAS-eQTL associations were identified and stored in AtMAD. For expression–methylation quantitative trait loci (emQTL), we identified 265 776 emQTLs and 122 344 pathway-mQTLs. For TWAS and EWAS, we obtained 62 754 significant phenotype-gene associations and 3 993 379 significant phenotype–methylation associations, respectively (Table 1). The schematic diagram of association network, which included genotype, gene, methylation, phenotype (including environment) and pathway, was shown in Figure 1B.

**Table 1.** Data summary for the AtMAD data repository in June 2020

Data type	Details	Total number of associations
cis-eQTL	eQTLs were defined if the SNP was within 1 Mb from the gene transcriptional start site (TSS)	11 796
trans-eQTL	eQTLs were defined if the SNP was beyond the region of 1 Mb from the gene transcriptional start site (TSS)	10 119
Environment-related eQTL	eQTLs that associated with at least one environmental factor	68 837
GWAS-related eQTL	eQTLs that related with known GWAS loci (collected from AraGWAS)	149 622
emQTL	emQTLs associated methylations and gene expression	265 776
Pathway-mQTL	Pathway-mQTLs connected methylations and pathways	122 344
Phenotype-pathway	Associations between pathways and phenotypes	1354
GWAS	GWAS identified variant-trait associations. Phenotypes were collected from AraPheno, GWAS information was obtained from AraGWAS Catalog with bonferroni threshold	44 636
TWAS	TWAS identified associations between gene expression and phenotype	62 754
EWAS	EWAS identified associations between DNA methylation levels and phenotypes	3 993 379

### Data mining and discovery

For this study, we mainly focused on the interrelations between different levels of genetic signals. By integrating data of genotypes, transcriptomes, methylomes and phenotypes for 620 accessions, we constructed a comprehensive population-based multi-omics association database, and revealed the potential bio-molecules which can explain phenotypic variations or biometabolic variations. For example, an intron eQTL (chr1-11315733-A-to-C) was significantly associated with 101 genes. Interestingly, AT1G31600 (TRM9) which contains this intron eQTL encodes a RNA-binding (RRM/RBD/RNP motifs) family protein, and the intron eQTL is predicted to affect the protein's structure (AT1G31600.2) by alternative splicing in SnpEff_v3.1 (29). One reasonable explanation is that the intron eQTL (chr1-11315733-A-to-C) changes the structure of TRNA METHYLTRANSFERASE 9 (TRM9) protein, which affects the binding ability of protein-RNA and then regulates a large number of downstream genes. Another upstream eQTL (chr2-9752742-G-to-T) of AT2G22920 (SCPL12) is highly connected to the elevation of habitats, and accessions with higher elevation show higher expression levels of SCPL12. The SCPL12 is recently discovered to be responsible for the production of saiginols and conferring greater UV light tolerance in plants (30). It suggests that this eQTL (chr2-9752742-G-to-T) is likely to influence the ultraviolet response of individuals by changing gene expression of SCPL12, and allow individuals to adapt to different elevations.

**Figure 2.**
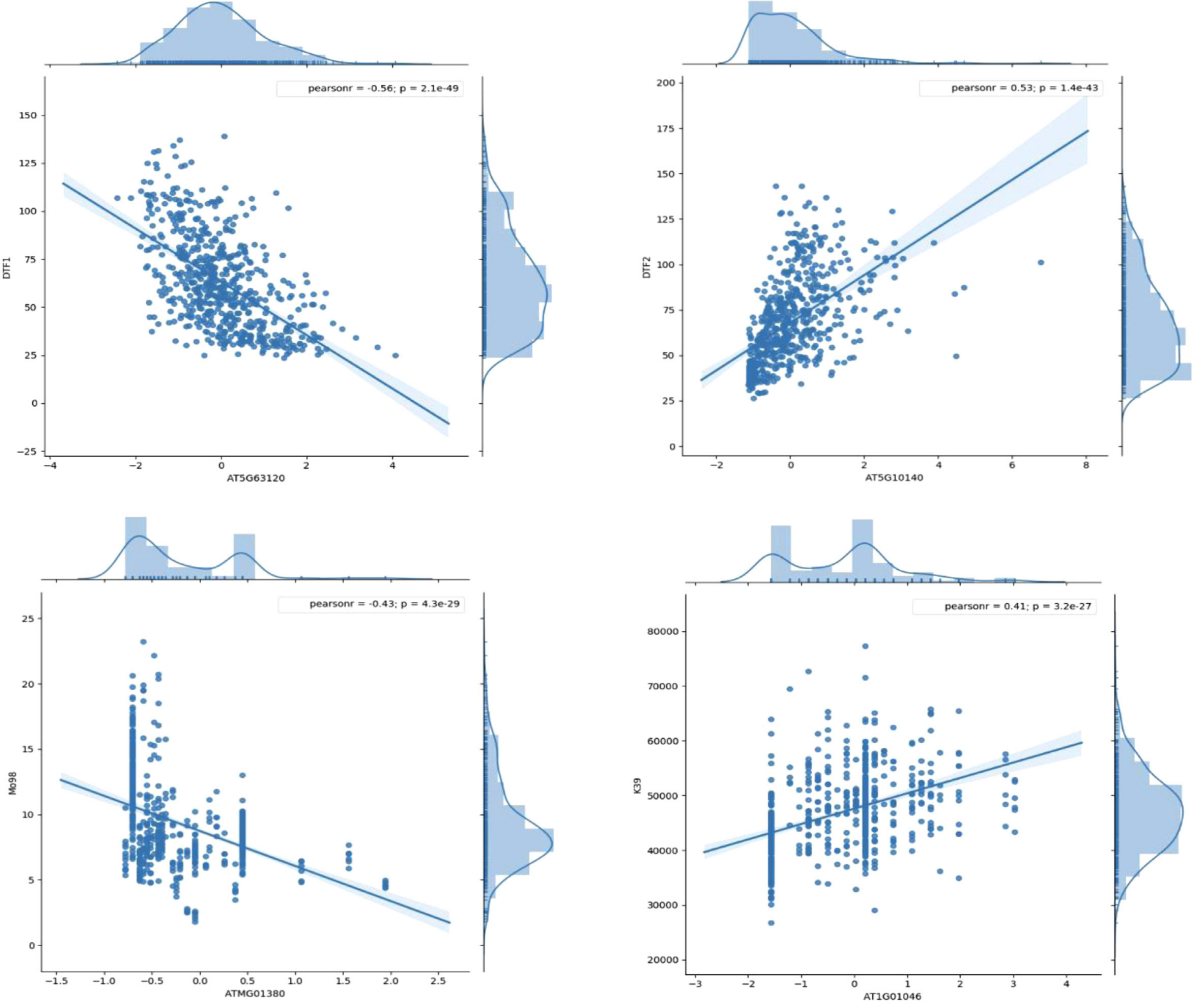
Four cases in the AtMAD. AT5G63120 (RNA HELICASE 30, RH30) and AT5G10140 (FLOWERING LOCUS C, FLC) were associated with DTF (Flowering time) respectively, which were confirmed by previous experimental evidences. ATMG01380 (RIBOSOMAL RNA5S, RRN5) and AT1G01046 (MIR838A) were linked to Mo98 (molybdenum concentrations in leaves) and K39 (potassium concentrations in leaves) significantly, which were unknown associations and identified for the first time in AtMAD.

By integrating meta-information of public phenotypes in AraPheno, we analyzed putative genes that are associated with each specific phenotype (Figure 2). In study 2 of AraPheno in 1001 project, a total of four genes (AT2G20440, AT5G10140, AT5G63120 and AT3G10010) were assigned to DTF spain 2008 (1st experiment), two of them AT5G10140 (FLC, Pearson = 0.6213, FDR = 0.005) and AT5G63120 (RH30, Pearson = −0.5833, FDR = 0.023) were previously inferred to be involved in flower development process with experimental supports (31,32). Moreover, AT5G08660 (PSI3) was identified to connect to YieldMainEffect2009 (seed weight) in AtMAD, which was consistent with the positive role of PSI3 in regulating plant growth and seed development (33). In AtMAD, many novel gene-phenotype links have also been identified, such as ATMG01380 (encoding mitochondrial protein) was significantly associated with Mo98 (molybdenum concentration in leaves), AT1G01046 (encoding a microRNA of unknown function) was connected to K39 (potassium concentrations in leaves). These gene–phenotype connections showed strong correlations in large sample sizes, and provided a reliable basis for further exploring the genomic function.

Compared to the information at single-omics level, the multi-omics association network can convey more information for specific phenotype or biomolecule. Integration of multi-omics data in the network can not only help us to accurately identify functional biomolecules, but also provide potential genetic mechanisms for interpreting the functional associations.

### Browsing and searching a large variety of integrated data

The web-based interface of AtMAD can be freely accessed at http://www.megabionet.org/atmad and allows users to browse, search and download data.

On ‘Browse’ module, users can browse data in nine different panels: ‘eQTL’, ‘Environment-related eQTL’, ‘GWAS-related eQTL’, ‘emQTL’, ‘Pathway-mQTL’, ‘GWAS’, ‘TWAS’, ‘EWAS’ and ‘Phenotype-pathway’. In ‘eQTL’, ‘Environment-related eQTL’, ‘GWAS-related eQTL’, ‘emQTL’ and ‘Pathway-mQTLs’ pages, we provided comprehensive information for reliable associations between genetic molecules at different levels. On ‘GWAS’, ‘TWAS’, ‘EWAS’ and ‘Phenotype-pathway’ panels, by clicking study ID, users can view the study information and query potential biomarkers which are associated with specific phenotype.

On ‘Search’ module, users can search AtMAD by genomic range, gene, phenotype and pathway. In the genomic range page, we provide detailed information about ‘eQTLs’, ‘emQTLs’, ‘Pathway-mQTLs’ and ‘GWAS loci’ which are within the set range. The gene page offers five different panels: ‘Summary’, ‘eQTLs’, ‘Meth-Exp correlation’, ‘TWAS’ and ‘direct gene-phenotype associations’. On the phenotype page, the information of ‘GWAS associations’, ‘TWAS associations’, ‘EWAS associations’, and ‘Pathway associations’ related to specific phenotype are presented in order. The pathway result page offers pathway-associated methylations and phenotypes. We also provide a vector diagram of boxplot or scatter plot to show the correlation between multi-omics data. By clicking the ‘network’, users can view a sub-network which comprises eQTLs, genes, methylations, pathways and phenotypes, all associations are connected by solid lines.

## SUMMARY AND FUTURE DIRECTIONS

Increasingly abundant multi-omics data (e.g. genome, transcriptome, methylome and phenome) for *A. thaliana* enables scientists to study the genetic mechanism of many complex traits. Based on large-scale population data, AtMAD describes and offers free access to substantially reliable associations among multi-omics of Arabidopsis, it integrates different data types (genomics, transcriptomics, methylomics and phenomics) and provides dedicated tools to explore them. AtMAD is the first database for multi-omics association analysis in Arabidopsis and makes it possible to discover plant biological processes with evidence at different levels. For the extensive associations, we identified a variety of known and novel associations, known associations, such as FLC and RH30 were associated with DTF (day to flowering), and PSI3 was connected to YieldMainEffect2009 (seed weight); previously uncharacterized associations, such as ATMG01380 (encoding mitochondrial protein) being significantly associated with Mo98 (molybdenum concentration in leaves) and AT1G01046 (encoding a microRNA of unknown function) being connected to K39 (potassium concentrations in leaves). These multi-omics association information provides us not only with further confirmation of previously inferred bio-associations, but also with new genetic evidence of specific biological processes. We believe that AtMAD provides valuable multi-omics association resources and will facilitate researches of plant genetics and functional genomics.

However, some limitations exist in the performed analyses. (i) In AtMAD, the association pairs obtained from GWAS or correlation statistics are correlation but not causation, the potential causative and adaptive relationships remain further validation. (ii) Considering the data types of TWAS, EWAS, emQTL, phenotype-pathway and pathway-mQTL, we used Pearson plus Spearman correlation analyses as previously described (24,25,34,35), which might be sensitive to population structure. (iii) Outliers (in gene expression, methylation or phenotypic data) are inevitable during data generation. Spearman correlation can significantly weaken the confounding effects of outliers among continuous data and then improve the reliability of results to certain extent. (iv) Although Bonferroni or Benjamini–Hochberg method was used in results correction, it should be aware that it is not possible to completely eliminate false positives in any GWAS or correlation analyses. (v) We detected eQTLs using package Matrix eQTL, of which correction method for population structure is different from mixed model. Comparing the results of both methods (Matrix eQTL (19) and mixed model GEMMA (22)), we noticed that positive results were similar but with slight difference between the two methods and the differences mainly exist in results with higher *P* value portion. Therefore, the eQTLs in AtMAD are overall reliable, but caution needs to be taken when using results with higher *P* value.

In the coming years, the 1001G+ project will continue to generate more genomes from a diverse collection of *A. thaliana* strains and annotate them with transcriptome and epigenome information. In 1001G+ project, large or complex structural variants, as well as simple variants inside complex variants will be discovered with the application of long-read sequencing. Based on this, we will continue to maintain and update the content in AtMAD by the following strategies: (i) including structural variants into our multi-omics associations. (ii) expanding genomes, transcriptomes and epigenomes for more Arabidopsis strains. (iii) integrating more public multi-omics dataset from Gene Expression Omnibus, Sequence Read Archive and other public sources besides 1001 Genomes. (iv) including more multi-omics dataset of different plant tissues (roots, flowers, etc.).
